# The spatial-temporal clustering of *Plasmodium falciparum* infection over eleven years in Gezira State, The Sudan

**DOI:** 10.1186/1475-2875-9-S2-P22

**Published:** 2010-10-20

**Authors:** Samia E Mirghani, Bakri YM Nour, Sayed M Bushra, Ibrahim El Hassan, Robert W Snow, Abdisalan M Noor

**Affiliations:** 1 University of Gezira, Blue Nile National Institute for Communicable Diseases, P.O.Box 101, Wad-Medani, Sudan; 2 Institute of Endemic Diseases, Department of Parasitology and Molecular Epidemiology, University of Khartoum, Sudan; 3 Malaria Public Health and Epidemiology Group, Centre for Geographic Medicine, KEMRI-University of Oxford - Wellcome Trust Collaborative Programme, Kenyatta National Hospital Grounds (behind NASCOP), P.O. Box 43640-00100, Nairobi, Kenya; 4 Centre for Tropical Medicine, Nuffield Department of Clinical Medicine, University of Oxford, CCVTM, Oxford OX3 7LJ, UK

## Background

Malaria infection and disease exhibit microgeographic heterogeneity which if predictable could have implications for designing small area intervention. Here we investigate the space-time clustering of *Plasmodium falciparum* infections using data from repeat cross-sectional surveys in Gezira State, a low transmission area in northern Sudan.

## Methods

Data from cross-sectional surveys undertaken in January each year from 1999-2009 in 88 villages in the Gezira state were assembled. During each survey, about a 100 children between the ages 2 to1 0 years were sampled to examine the presence of *P. falciparum* parasites. In 2009, all the villages were mapped using global positioning systems. Cluster level data were analysed for spatial-only and space-time clustering using the Bernoulli model and the significance of clusters were tested using the Kulldorff scan statistic.

## Results

Over the study period, 96,022 malaria slide examinations were undertaken and the *P. falciparum* prevalence was 8.6% in 1999 and by 2009 this had reduced to 1.6%. The cluster analysis showed the presence of one significant spatial-only cluster in each survey year and one significant space-time cluster over the whole study period. The primary spatial-only clusters in 10/11 years were either contained within or overlapped with the primary space-time cluster.

## Conclusion

The results of the study confirm the generally low malaria transmission in the state of Gezira and the presence of spatial and space-time clusters concentrated around a specific area in the south of the state. Improved surveillance data that allows for the analysis of seasonality, age and other risk factors need to be collected to design effective small area interventions as Gezira states targets malaria elimination.

